# Dentists' Ability to Identify Tooth Resorption on Radiographic Images and Their Preferences for Terminology

**DOI:** 10.1111/adj.70003

**Published:** 2025-08-20

**Authors:** Sheema Pham, Paul V. Abbott

**Affiliations:** ^1^ UWA Dental School The University of Western Australia Nedlands Western Australia Australia

**Keywords:** classification, inflammatory resorption, invasive resorption, replacement resorption, terminology, tooth resorption

## Abstract

**Background:**

Tooth resorption has no universal classification, which leads to confusion. The aims were to assess dentists' ability to radiographically identify resorption and to determine their terminology preferences for three types of resorption.

**Materials and Methods:**

Dentists completed an electronic questionnaire. Part 1 concerned professional profiles plus self‐rating of each participant's level of resorption knowledge. In Part 2, participants identified types of resorption from radiographs. In Part 3, participants chose their preferred terminology and provided reasons for their choices.

**Results:**

Of 444 complete responses, 55.6% of participants self‐rated their knowledge as ‘Acceptable’. The average number of correct responses when identifying resorption was 52.9% (6.3 out of 12; range: 0–12). Significant differences existed for gender, practice area, graduation year and education level. Preferred terms were external inflammatory resorption (72.3%) and external replacement resorption (63.3%) but no clear preference existed for external invasive resorption (31.8%) or external cervical invasive resorption (35.6%). Most common reasons for selecting terms were ‘more descriptive’, and a ‘more accurate representation of what occurs in the tissues’.

**Conclusion:**

On average, dentists correctly identified 52.9% of the resorptive defects. There is a need for a standardised classification of the different types of tooth resorption.

**Summary**

- There are at least 17 different classifications of tooth resorption in the dental literature which have caused confusion amongst dentists.
- This survey confirmed this confusion when dentists were asked to identify different types of resorption based on their radiographic appearances.
- These results confirm the need for a universal classification of tooth resorption, and they also demonstrate the need for further education and training.
- The participating dentists provided their preferences of terminology for external inflammatory and external replacement resorption, but there was no clear preference of terminology for external invasive resorption.

## 1 Introduction

Identifying and understanding a disease or condition are the initial steps towards formulating a diagnosis, which is a pre‐requisite for any treatment in medicine and dentistry. Tooth resorption can be physiological or pathological in nature, and knowledge of the processes is required to appropriately diagnose and manage the condition. There are 11 different types of tooth resorption according to the most recently published classification [1]. They are broadly divided into internal and external resorption. Internal resorption is further divided into surface, inflammatory and replacement resorption, whereas external resorption is classified as surface, inflammatory, replacement, invasive, pressure, orthodontic, physiological and idiopathic [1]. However, there have been 17 different classifications of the various types of tooth resorption published in the literature from1970 to 2022 [2]. Various terms have been used for the same type of resorption, and this incongruity in terminology is likely to lead to confusion amongst practitioners.

General dentists are often the patient's first point of contact when it comes to routine and emergency dental care. Therefore, their ability to identify the state of the teeth and associated structures is of paramount importance. A retrospective analysis of data from a referral‐based specialist endodontic practice found that there were often inconsistencies between the diagnosis and primary reason for the patient referral, and the actual diagnosis and treatment carried out after assessment by the endodontist [3]. For example, 3.9% of the patients were referred for the management of tooth resorption, but this was less than half the actual number noted (8.65%) to have resorption after assessment and diagnosis by the endodontist [4]. Although 8.65% may be considered to be a low number, it reflects a relatively high proportion of the referred patients where adverse outcomes could have occurred due to the lack of a correct diagnosis with resultant inappropriate treatment. Unpublished data from the same author indicated that 36% of the patients referred for the management of internal inflammatory resorption actually had external invasive resorption (P. V. Abbott, personal communication).

Although many forms of tooth resorption can be identified with the use of CBCT imaging, the initial identification of tooth resorption is more likely to be via routine intra‐oral radiographs such as periapical and/or bitewing radiographs. Once a dentist identifies a resorptive condition, a choice can then be made as to whether further radiographs from different angulations and/or a CBCT image need to be obtained for further assessment of the resorption. Each type of resorption has specific radiographic features which distinguish it from the other types [1]. Other information—such as the clinical findings, additional radiographs and/or CBCT images—are useful aids to determine the extent of the resorption, the surfaces involved, and whether the pulp is involved. This extra information is also useful for determining the management options and prognosis of the tooth. Not all resorptive defects will require a CBCT image, and although there has been a 204.3% increase in the number of CBCT machines in Australia in the 6‐year period from 2014 to 2020 [5], there are many dental practices that do not have CBCT machines—for example, in 2020 there were 706 CBCT machines in Australia [5] compared with 7169 dental practices [6]. Hence, the majority of dentists may not have access to a CBCT machine at the time that a patient presents for an examination. Therefore, it is essential that dentists have the ability to diagnose the different types of resorption from 2‐dimensional radiographic images.

Part of the misunderstanding related to tooth resorption may be due to the various classifications. Lin et al. [2] reviewed the evolution, rationales and controversies of tooth resorption and identified the shortfalls within previous nomenclature used to describe the various types of resorption. They highlighted that the biggest issue was potential miscommunication that may occur between clinicians, teachers, students and researchers [2]. As the clinical management of each type of resorption is different, miscommunication and confusion may lead to negative outcomes or inappropriate treatment for patients [2]. There have been different approaches taken by authors when developing their classifications, and Lin et al. [2] categorised them as based on causation, management, site, or pathological process. Specificity and clarity are important, and this led to the classification proposed by Abbott and Lin [1] which identifies conditions based on what is occurring in the tissues—that is, the type of pathological or physiological process. Their classification scheme follows the key principles of being possible to use clinically, is meaningful, useful, unambiguous and universally applicable [1, 7, 8].

A survey conducted in 2018 revealed that the terminology used to describe various dental conditions influenced the treatment decisions made by dentists as to whether or not they would treat the tooth [9]. For example, when the term ‘chronic’ was used instead of ‘asymptomatic’, the dentists were significantly more likely to treat the presenting condition [9]. This study highlights that terminology is important and it also emphasises the need to have clear terminology in any classification of diseases or conditions.

Over the last two decades, terms for resorption have often included the word ‘‐related’ within classifications (e.g., ankylosis‐related resorption, infection‐related resorption, and so forth) [10]. However, these terms also do not identify the pathological or physiological processes that are occurring in the tissues. They indicate something which may be present or associated with the resorption, but there may be other factors associated with the resorption which are not mentioned with this approach. Hence, this approach is inappropriate as well as inconsistent since it cannot be applied to all types of tooth resorption [1]. It is also not consistent with other classifications of diseases or conditions in dentistry or medicine.

The aims of this study were to assess dentists' ability to identify different types of resorption based on their radiographic appearance and to determine whether dentists have preferences for particular terminology to describe three resorptive conditions that have various terms in the literature.

## 2 Materials and Methods

Following approval from the University of Western Australia's Research Ethics Committee, in accordance with the requirements of the National Statement on Ethical Conduct in Human Research and the policies and procedures of the University of Western Australia (Approval No. 2021/ET000464), an electronic questionnaire using the Qualtrics Survey Software (Provo, UT, USA; Version 07.2021) was developed and sent via email to general and specialist dentists across Australia. The survey was constructed by a postgraduate endodontic registrar and a senior endodontist. It was tested for clarity by another senior endodontist and two postgraduate endodontic registrars who suggested minor changes to the initial text. The correct radiographic diagnosis of the type of resorption in each image was also assessed by reviewing the responses of each assessor during the review process. The final questionnaire was sent via email to general and specialist dentists across Australia. This software enabled participation to be anonymous. The Australian Dental Association (Western Australian Branch) and the Australian Society of Endodontology (ASE) distributed the questionnaire to their members via their email addresses and via their members‐only Facebook groups. Members of other professional‐based Facebook groups (such as Dental Product Review ‘DPR’) and social media platforms were also invited to participate in the survey. In order to obtain a margin of error of 5% from 19,396 registered dentists in Australia (Dental Board of Australia/Australian Health Practitioner Regulation Agency [11], dated 28 July 2022) with a 95% confidence interval, at least 377 responses were required. This was calculated using a statistical tool (https://www.qualtrics.com/blog/calculating‐sample‐size/) [12].

The survey consisted of three parts. The first two parts were based on surveys conducted by Hartmann et al. [13] and Jadav and Abbott [14]. The first part included questions to determine the sociodemographic and professional profiles of the participants—such as age, gender, level of experience, registration status (specialist or general), principal place of practice (very remote, remote, outer regional, inner regional or major city—according to the Australian Bureau of Statistics definitions of ‘Remoteness Areas’ [15]), and their self‐assessed knowledge of tooth resorption (‘low’, ‘acceptable’, ‘good’ or ‘very good’). The second part of the survey had 12 multiple choice questions where a radiograph was provided and the participants were asked to identify the type of tooth resorption from a list of possible answers. The radiographs used in this survey were taken from the senior author's collection of cases from his private and university clinical settings. The third part of the questionnaire had three questions where participants were asked to indicate their preferred terms for three types of external resorption—namely, ‘external inflammatory resorption’ or ‘external infection‐related resorption’; ‘external replacement resorption’ or ‘ankylosis‐related resorption’; and ‘external invasive resorption’, ‘external cervical resorption’ or ‘external invasive cervical resorption’. Participants were also asked to provide the reason(s) for their choice of terminology using free text answers. File S1 contains all the survey questions and the correct answers to the questions in the second part of the survey.

The level of knowledge of participants in the second part of the survey was assessed using a scoring system where each correct answer was assigned one point, and incorrect answers had a score of zero. Hence, the maximum possible score was 12 points and the minimum possible score was zero. Scores were considered to indicate the actual level of a participant's knowledge as Low (0–3), Acceptable (4–6), Good (7–9) or Very Good (10–12) as per Hartmann et al. [13] and Jadav and Abbott [14].

Responses for each preferred term for part three of the survey were reported as frequencies. The reasons for the dentists' choice of preferred terminology were sorted into seven common categories. These were ‘No reason’, ‘Familiar’, ‘Descriptive’, ‘Self‐explanatory’, some variation of ‘Generalised’ or ‘Aetiology/Location specified’ (dependent on the specific term) and ‘Other’.

Analysis of the results was primarily completed using descriptive statistics and the use of Excel 2016 MSO (16.0.4549.1000) 64‐bit (Microsoft, Redmond, WA, USA). Following this, statistical analysis was completed with IBM SPSS STATISTICS version 28 software (IBM Corp, Armonk, NY, USA). Mean scores of participants according to their self‐reported knowledge, area of work, and gender were assessed with the application of one‐way ANOVA and Tukey's post hoc test. An independent samples *t*‐test was used to test the association between registration status (specialist or general dentist). The *α*‐level was set at 0.05.

## 3 Results

A total of 504 responses were received, of which 60 had incomplete responses and were deleted from the analysis. Hence, 444 participants provided valid answers for all three parts of the survey. Table 1 summarises the demographic details and the participants' self‐reported level of knowledge of resorption.

**TABLE 1:** Summary of demographic data and participants' self‐reported level of knowledge of tooth resorption.

Participants' responses	Number (%)	Mean score ± SD	*p*
*Gender*			
Male^a^	201 (45.4)	6.87 ± 2.51	< 0.001
Female^b^	231 (52.1)	5.95 ± 2.17	
Prefer not to disclose^ab^	8 (1.8)	5.75 ± 1.49	
Prefer to self describe^ab^	3 (0.7)	6.67 ± 2.08	
*Year of primary dental qualification*			
Prior to 1951^1^	1 (0.2)		0.002
1951–1960^1^	1 (0.2)		
1961–1970^1^	0 (0.0)		
1971–1980^ab^	7 (1.6)	7.43 ± 3.10	
1981–1990^a^	25 (5.6)	7.64 ± 2.33	
1991–2000^ab^	24 (5.4)	7.29 ± 3.00	
2001–2010^ab^	77 (17.3)	6.59 ± 2.65	
2011–2020^b^	309 (69.6)	6.11 ± 2.17	
*General dentist or dental specialist*			
General dentist	386 (86.9)	6.12 ± 2.22	< 0.001
Dental specialist	58 (13.1)	7.98 ± 2.65	
*Place of work*			
RA1: Major cities of Australia^a^	341 (76.8)	6.42 ± 2.32	0.014
RA2: Inner regional Australia^a^	74 (16.7)	6.38 ± 2.53	
RA3: Outer regional Australia^a^	24 (5.4)	6.17 ± 2.18	
RA4: Remote Australia^1^	0 (0.0)		
RA5: Very remote Australia^b^	5 (1.1)	3.00 ± 1.23	
*Self‐reported knowledge*			
Low^a^	105 (23.7)	5.89 ± 2.06	< 0.001
Acceptable^a^	250 (56.5)	5.97 ± 2.09	
Good^b^	56 (12.6)	7.07 ± 2.72	
Very good^c^	32 (7.2)	9.75 ± 1.50	

*Note:* There are significant differences between the mean values denoted by various superscript letters (*p* < 0.05).

^1^ Responses were excluded in the one‐way ANOVA Tukey's post hoc test as they had fewer than two cases.

**FIGURE 1:**
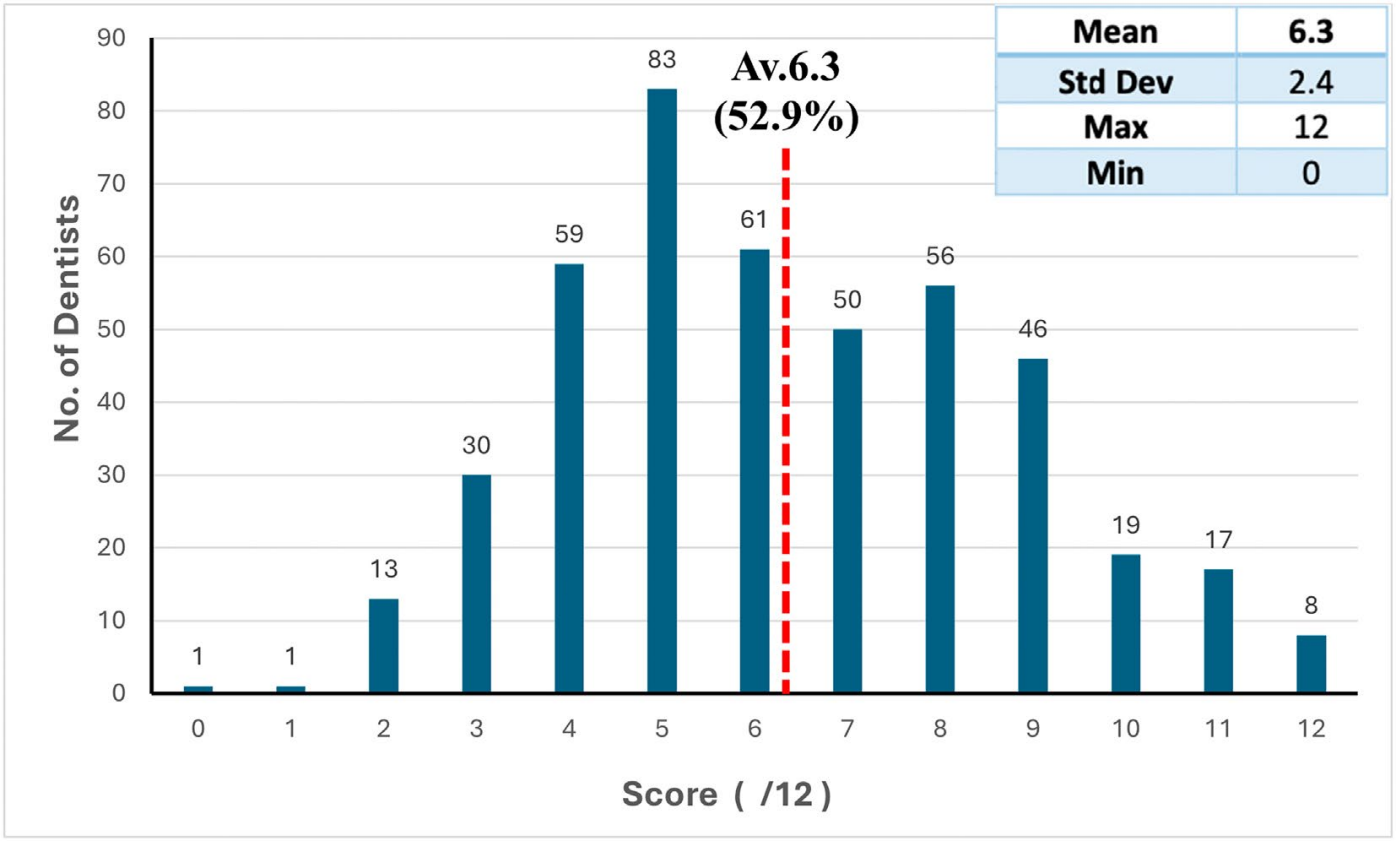
The number of dentists and the number of correct responses.

In Part 2 of the survey, the average number of correct answers per participant was 6.3 out of a possible maximum score of 12, with a range of 0–12 correct answers (Figure 1). The average number of correct answers for the 12 questions was 52.9%, with a range of 22.7%–88.1% (Table 2). Internal inflammatory resorption had the highest percentage of correct answers (87%); external replacement resorption had the lowest (33%) percentage of correct answers (Table 3).

**TABLE 2:** The number of participants who correctly identified the type of resorption in each question.

Question	No. of correct answers (*n* = 444)	Percentage
Case 1	168	37.8
Case 2	183	41.2
Case 3	308	69.4
Case 4	385	86.7
Case 5	308	69.4
Case 6	101	22.7
Case 7	165	37.2
Case 8	391	88.1
Case 9	236	53.2
Case 10	242	54.5
Case 11	146	32.9
Case 12	186	41.9
Average	234.9	52.9

**TABLE 3:** The number of correct answers and the possible total scores for each type of resorption.

Resorption type	No. of correct answers	Possible total	% Correct
Internal inflammatory	385	444	87
External replacement	146	444	33
External inflammatory^a^	885	1332	66
External invasive^a^	747	1776	42
Orthodontic	308	444	69
Physiological	165	444	37
Pressure	183	444	41
Totals	2819	5328	53

^a^ External inflammatory resorption and external invasive resorption had 3 and 4 questions, respectively. Hence, the possible totals are 3 × 444 = 1332 and 4 × 444 = 1776, respectively.

Statistical analysis revealed that there was a significant relationship between the mean scores (*x̄*) achieved and the dentists' self‐reported level of knowledge. Those who selected ‘very good’ as their self‐reported level of knowledge of resorption had higher mean scores (*x̄* = 9.75), and this was significant (*p* < 0.001) when compared to all other groups. Those who selected ‘good’ as their self‐reported level of knowledge (*x̄* = 7.07) also performed significantly better compared with those who rated their knowledge as ‘low’ (*p* = 0.005) and ‘acceptable’ (*p* = 0.003). The mean scores of the ‘low’ and ‘acceptable’ groups were not significantly different (*p* = 0.986) (*x̄* = 5.89 and *x̄* = 5.97, respectively).

There was a statistically significant association between the mean scores when sorted according to the area of work. Those who worked in ‘Very Remote Australia’ had lower mean scores (*x̄* = 3) compared to all other groups as follows: ‘Major cities of Australia’ (*p* < 0.007) (*x̄* = 6.42), ‘Inner Regional Australia’ (*p* = 0.010) (*x̄* = 6.38) and ‘Outer Regional Australia’ (*p* = 0.031) (*x̄* = 6.17).

The decade of graduation had a statistically significant effect on mean scores. Those who graduated in 1981–1990 attained the highest mean score (*x̄* = 7.64), and those who graduated in the years 2011–2020 had the lowest mean score (*x̄* = 6.11) (*p* < 0.015). There was no significant difference between any other decades of graduation. The groups ‘Prior to 1951’ and 1951–1960 had only one participant each and there were no participants from 1961 to 1970—hence, these groups were not included in the analysis of scores related to the decade since graduation.

Gender had a very high statistical significance, with males having a higher mean score (*x̄* = 6.87) than females (*x̄* = 5.95) (*p* < 0.001). There was no significant relationship between the other groups.

Specialists had significantly higher mean scores (*x̄* = 7.98) compared with general dentists (*x̄* = 6.12) (*p* < 0.001). Endodontists made up the majority of the specialist group (60%) and their mean score was 9.6 (Table 3).

The terms ‘external inflammatory resorption’ and ‘external replacement resorption’ were clearly preferred by the majority (72.3% and 63.3% respectively) of participants (Table 5). However, the terms ‘external invasive resorption’ and ‘external cervical invasive resorption’ were similar in preference (31.8% and 35.6%, respectively) (Table 5).

**FIGURE 2:**
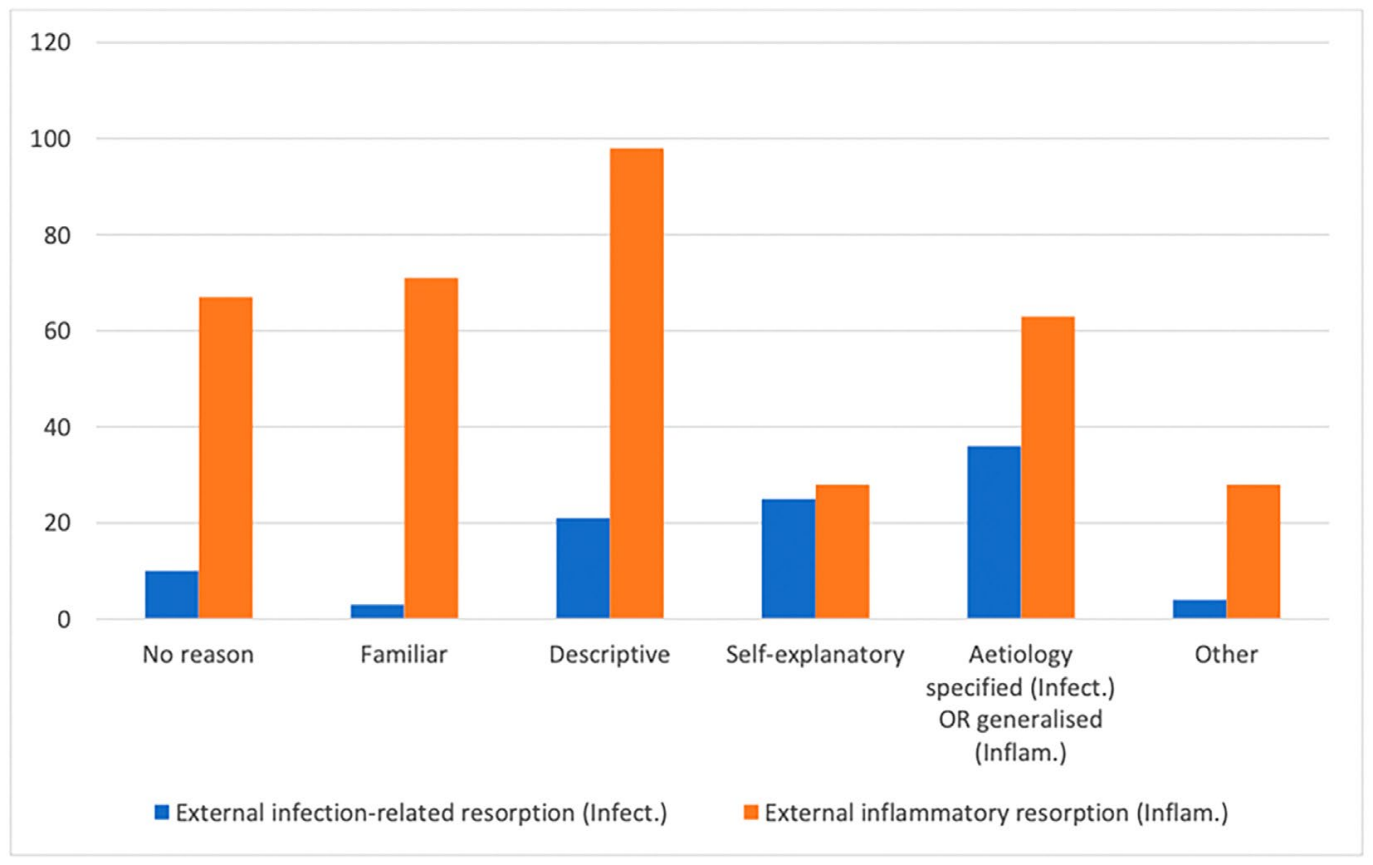
Summary of the reasons for selecting the term ‘external inflammatory resorption’ versus ‘external infection‐related resorption’.

**FIGURE 3:**
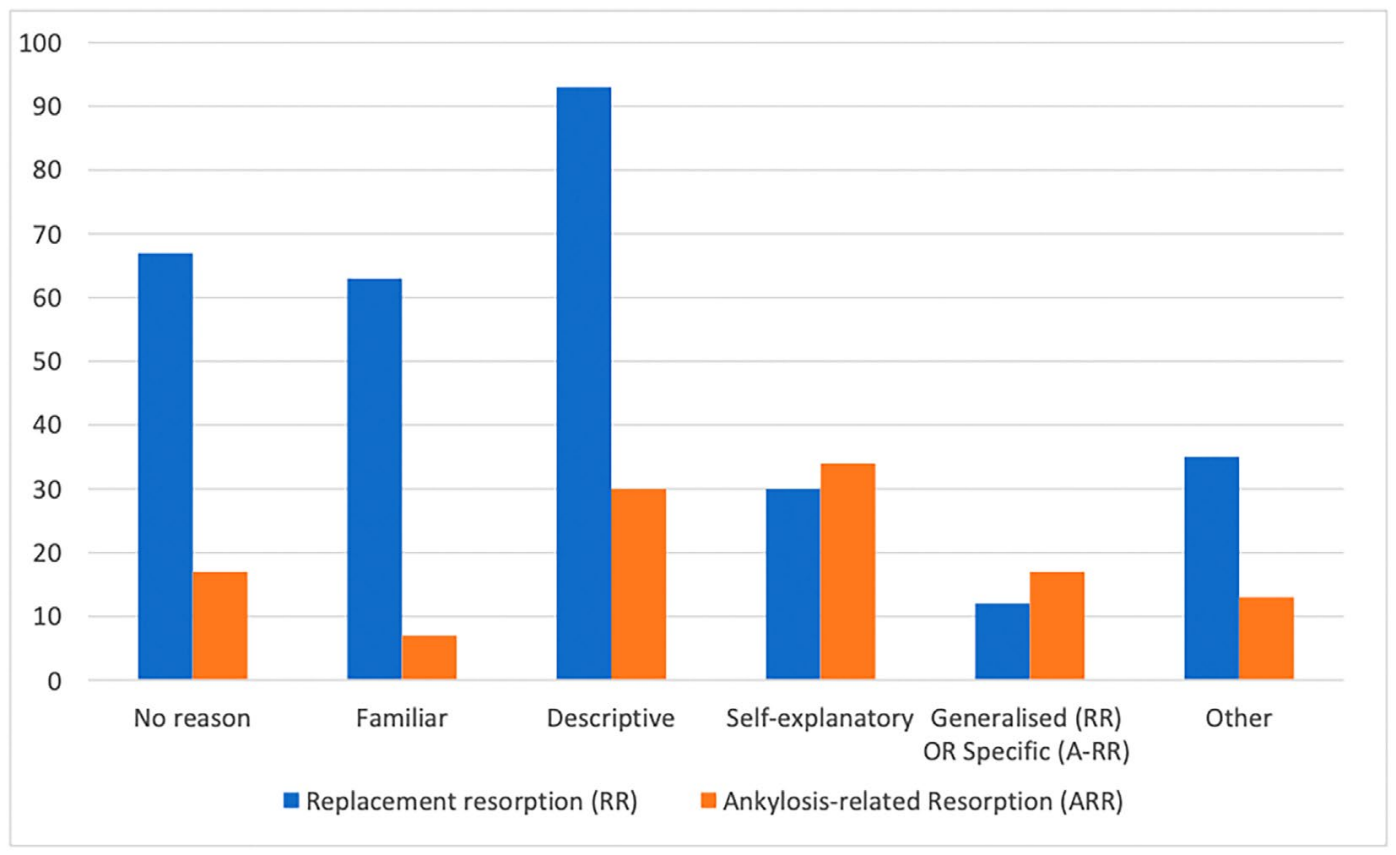
Summary of the reasons for selecting the term ‘external replacement resorption’ versus ‘external ankylosis‐related resorption’.

Many reasons for choosing their preferred terminology were provided by the participants (File S2). The most common reason for selection of the term ‘external inflammatory resorption’ was that it was more descriptive or an accurate representation of the condition, whereas the main reason for selecting ‘external infection‐related resorption’ was that it specified the cause of the resorption (Figure 2). ‘External replacement resorption’ was also selected predominantly because it was more descriptive of what is occurring in the tissues, whereas those that selected ‘external ankylosis‐related resorption’, much like external infection‐related resorption, preferred that the aetiology was included within the name (Figure 3).

**FIGURE 4:**
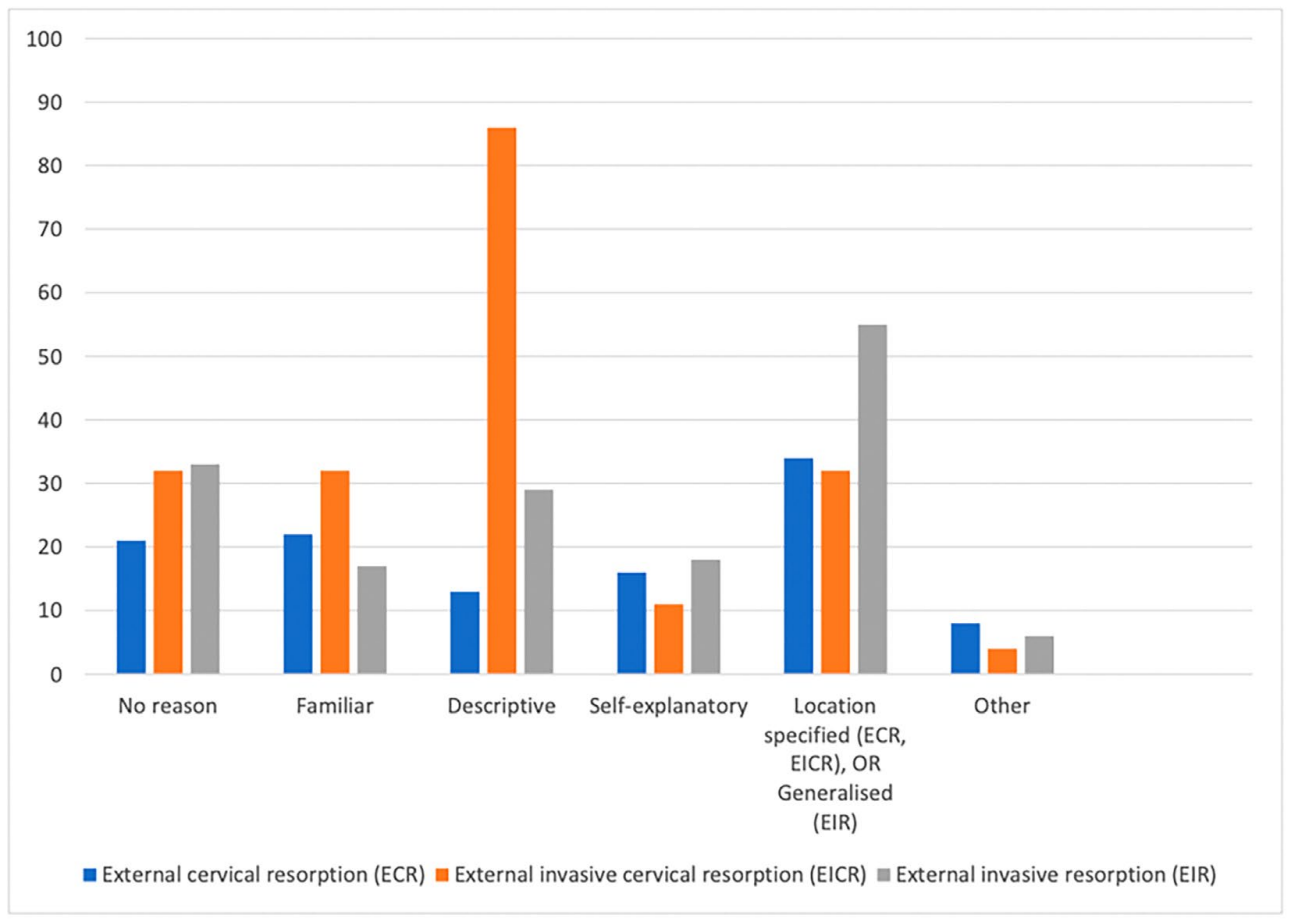
Summary of the reasons for selecting the terms ‘external invasive resorption’, ‘external invasive cervical resorption’ and ‘external cervical resorption’.

When considering the term ‘external invasive resorption’, the most common reason indicated by participants was that it was broader and that it should not account for the location because it does not always occur in the cervical region of the tooth (Figure 4). Of the participants that selected ‘external cervical resorption’, specification of the location was a key reason for this group. Participants who chose ‘external invasive cervical resorption’ stated that they thought it was the most descriptive term for this condition.

## 4 Discussion

The average score in Part 2 of this study was 52.9% (6.3 out of 12) which is considered acceptable in most educational settings. However, when diagnosing diseases or conditions, this score implies that, on average, 1 in 2 conditions was misdiagnosed. The scores ranged from 0 to 12 correct responses, meaning there were some participants who were unable to correctly identify the type of resorption in any question.

External invasive resorption and internal inflammatory resorption were often misdiagnosed by participants of this survey. This is consistent with data from a referral‐based specialist practice, where more than one third of the patients referred for the management of ‘internal inflammatory resorption’ instead had external invasive resorption (Abbott, personal communication). A small minority selected the answer ‘no resorption but caries is present’ which is also consistent with other conditions that external invasive resorption is typically confused with.

The highest number of correct answers was provided for internal inflammatory resorption, with 87% of participants correctly selecting this response (Table 3). There were more questions (4) regarding external invasive resorption, which may have given participants more of an opportunity to answer this question correctly but only 44% of these had selected the correct answer. On the other hand, having more questions about this type of resorption provided more opportunity for participants who confuse this condition to incorrectly identify these cases as internal inflammatory resorption, and ‘by chance’ increase their odds of ‘guessing’ internal inflammatory resorption on the one radiograph that truly represented this condition. Both the (correctly identified) internal inflammatory resorption and the external invasive resorption cases which were incorrectly diagnosed were ‘ovoid’ in shape and not necessarily at the cervical region of the tooth, which may have been the contributing factor to why participants thought they were all internal inflammatory resorption. Perhaps if the terminology was taught as ‘external invasive resorption’ rather than by its counterpart terms (external cervical resorption and external invasive cervical resorption) which contain the possible location of the defect, participants may have considered external invasive resorption for these two questions which were incorrectly diagnosed.

The condition that was least likely to be diagnosed correctly was external replacement resorption. This condition was also confused with external inflammatory resorption (lateral). This result shows that there is also generally a poor knowledge of the radiographic appearances of external inflammatory resorption (lateral) and external replacement resorption. Within the choice of possible conditions provided for the tooth with external inflammatory resorption, the term ‘external infection‐related resorption’ was provided as one of the choices. This may have confused some participants if they were not familiar with this term being another name for ‘external inflammatory resorption’.

The high correlation between self‐rated knowledge and correct responses in the survey was a significant finding of this study and agrees with Hartmann et al. [13] and Jadav and Abbott [14]. This may be reflective of the personality type of dentists, or perhaps the personality type of dentists that were inclined to complete the survey and hence formed the sample group. A study of the personality type of dental graduates conducted over 20 years from 1964 to 1984 using the Myers‐Briggs Type Indicator reported that the majority of participants were categorised as having either introversion (I), sensing (S), thinking (T) and judgement (J) (ISTJ) or extraversion (E), sensing (S), thinking (T) and judgement (J) (ESTJ) personality types [16]. A similar study of dental specialists indicated that more than 50% of the individuals tested had IST personality types [17]. The common ‘sensing, thinking and judgement’ characteristics suggest an organised analytical personality type, with a need for facts/details, based on reason, that may mean they are more self‐critical and honest about their level of knowledge. Although the Myers‐Briggs Type Indicator is a popular personality test, it has been reported to have several flaws as it is not in agreement with known data and facts. Attempts to dichotomise some aspects of personality that are not true opposites, makes it difficult to test validity through self‐verification, and testability is limited due to the inherent avoidance of strong statements [18]. Hence, this justification should be considered with caution.

Participants practicing in ‘Very Remote Australia’ had the poorest scores. Many types of resorption can be associated with dental trauma (e.g., external inflammatory resorption, external replacement resorption, external invasive resorption and internal inflammatory resorption) and require follow up examination, or may be noted as incidental findings as part of routine examinations. Due to travel considerations and patient attitudes or motivation towards preventive dental care in remote cities or towns, patients may not follow conventional preventive protocols, and these patients may only seek emergency dental treatment. This may justify the poor knowledge of dentists due to low exposure to these conditions. This finding is similar to that of Jadav and Abbott [14], who reported that city dentists had marginally higher mean scores for the management of dental trauma compared to rural dentists.

The results showed a significant relationship between the number of years since graduation from dental school and the mean scores. The two groups that were significant in terms of this relationship were the dentists that graduated between 1981 and 1990 and the most recent graduates (2011–2020). This is similar to the results reported by Jadav and Abbott [14] but contrasts with the results of Hartmann et al. [13] who found the highest scores were for dentists with 10–19 years of experience. They attributed this to generational advantages such as improved utilisation of web‐based learning—for example, the International Association of Dental Traumatology web‐based guidelines (https://www.iadt‐dentaltrauma.org) [19] and the Dental Trauma Guide's web‐based program (https://dentaltraumaguide.org) [20]. In this group, the dentists with more years of experience may have treated more cases, they may have been exposed to more conditions, or perhaps their university programs were structured differently, enabling better learning outcomes.

There was a significant increase in mean scores for registered specialists compared to general dentists. Hartmann et al. [13] showed that of the specialties, endodontists had the strongest grasp on the management of traumatic dental injuries. If there had been a larger representation of other specialties in this current study, it is highly likely that the same conclusion may have been drawn. Endodontists formed the majority of the specialist group in the current study, and their average score was 9.6 (Table 4). This was no surprise given that endodontics is the specialty most involved with the diagnosis and management of the various types of resorption. However, it was surprising that not all of the endodontists self‐reported their knowledge as ‘very good’, and not all of them achieved scores that could be considered as an indicator of ‘very good’ knowledge. Unfortunately, due to the under‐representation of other specialties, it was not possible to analyse each specialty separately, so all specialties were grouped as one. Hartmann et al. [13] also found that significantly better results were obtained when the participants had a higher degree (Masters and/or PhD) which is consistent with the specialist group scoring a significantly higher mean score than general practitioners in the current study.

**TABLE 4:** Endodontists' self‐rated knowledge of tooth resorption and a summary of their scores when assessing the 12 radiographs in Part 2 of the study.

Self‐rating	No. of endodontists	% Of endodontists
Low	1	2.9
Acceptable	1	2.9
Good	9	25.7
Very good	24	68.6
Total	35	100
Average score (± SD)	9.6 ± 1.3	
Range of scores (mode)	9–12 (9)	

**TABLE 5:** Summary of the respondents' preferred terminology for three types of external resorption.

Preferred term	No. of responses	%	Comment
*Qn 1. External inflammatory resorption vs. external infection‐related resorption*			
External inflammatory resorption	321	72.3	Large majority
External infection‐related resorption	96	21.6	
No response	27	6.1	
Totals	444	100	
*Qn 2. External replacement resorption vs. external ankylosis‐related resorption*			
External replacement resorption	281	63.3	Large majority
External ankylosis‐related resorption	121	27.3	
No response	42	9.5	
Totals	444	100	
*Qn 3. External invasive resorption vs. external cervical resorption vs. external invasive cervical resorption*			
External invasive resorption	141	31.8	Approx. equal
External invasive cervical resorption	158	35.6	Approx. equal
External cervical resorption	103	23.2	
No response	42	9.5	
Totals	444	100	

*Note:* The highlighted rows indicate the preferred term for each type of resorption.

Although the sample size in this study (444) was adequate in terms of achieving adequate statistical power, if more dentists had participated, there may have been a more accurate representation of the current ability of dentists to identify resorption on radiographs, shifting the data in either direction. Due to the manner in which the survey was distributed, it is not possible to know how many dentists actually received the invitation to participate; therefore, the percent of respondents cannot be calculated. However, the number of respondents was more than the number required (377) according to the power analysis. Additionally, since the survey was ‘voluntary’ there may also have been a degree of voluntary response bias—that is, dentists with an interest in tooth resorption may have been more inclined to take the survey.

Participants indicated their preferences for certain terms regarding tooth resorption and most provided reasons for their choices. The most common reason for the term ‘external inflammatory resorption’ was that it was more descriptive or a more accurate representation of the condition which is consistent with the guidelines regarding choice of terminology outlined by Abbott and Lin [1]. Conversely, the main reason for selecting ‘external infection‐related resorption’ was that it specified the cause of resorption in the name (Figure 2). In order to be consistent with classifications of other medical and dental conditions, diagnostic terms should not contain the aetiology, but they should represent what is happening in the tissues [1]. Within the reasons provided, a notable response included ‘I have now realised how little I know about the terms and types of resorption’ while other participants left no reason or multiple possible reasons as to why they preferred a particular term and one participant went on to say ‘I don't like either [terms]. This survey makes me think I have been using the wrong terms’.

The most common reason for selection of the term ‘external replacement resorption’ was also that it was more descriptive of what is occurring in the tissues, which is again consistent with the literature, whereas those that selected ‘ankylosis‐related resorption’ preferred this term because they felt it was self‐explanatory (Figure 3). Some participants who chose ‘external replacement resorption’ said they had ‘No idea what either of these terms are’ or that they ‘Prefer neither as not familiar with either’. This highlights the confusion within the profession which may well be related to the various terms that have been used in the literature. Dentists should be aware that ankylosis will always be present when teeth are undergoing external replacement resorption, but external replacement resorption will not necessarily be present during the initial ‘ankylosis’ phase [1, 21]. Given that these terms (ankylosis and replacement resorption) are not interchangeable because they represent different tissue reactions, ‘external replacement resorption’ is more descriptive of what is occurring in the tissues, and to maintain consistency, this term should be used.

When considering external invasive resorption, the most selected reason was that it was a broader term and it did not account for the location, with many participants also stating that it does not always occur in the cervical region (Figure 4). One participant stated ‘If it occurs elsewhere on the tooth the term cervical may cause confusion’ as their reason for choosing ‘external invasive resorption’ as their preferred term. Of the participants that selected ‘external cervical resorption’, the specification of the location was a key reason for their choice. When ‘external invasive cervical resorption’ was chosen as the preferred term, the main reason given was that it was the most descriptive term for the condition. One participant stated it ‘Relates to position and aggressiveness’. Given that this type of resorption does not always occur in the cervical region of the tooth, even though it often starts cervically (but it can then spread in any direction), it can be confusing from a diagnostic perspective [1]. The location of a condition should not be mentioned within the classification unless it always occurs in this position. The invasive nature of this type of resorption is well depicted by this term and so it should be kept in this classification to differentiate it from other forms of external resorption, particularly if the word cervical is omitted [1].

There was a variety of reasons for preferences which suggest that tooth resorption is a particular area of dentistry that may be deficient in the way it is taught and hence understood. On the one hand, many of the reasons provided for the preferred terms indicate that many dentists are aware of what a classification should entail and why some terminology should be preferred over others, which may help dentists to accept a more comprehensive and accurate classification for tooth resorption. However, on the other hand, incorrect responses or some of the reasons provided for preferred terms have brought to light the lack of knowledge about tooth resorption, plus possible negative attitudes towards change. These may be obstacles faced when attempting to standardise the teaching of resorption with a universal classification.

Attitudes towards standardising diagnostic terminology have been positive in dentistry [22]. Studies of medical cohorts are also in agreement on accepting changes to clinical practice guidelines when they are evidence‐based, with doctor's knowledge or experience and their perceived feasibility being common barriers to implementing such changes [23]. Some participants in the current survey appeared to have a good understanding of classifications, as demonstrated by some of the reasons for selecting ‘external inflammatory resorption’—such as ‘Good to standardise the terminology used in other literature’ or ‘Well‐established term that is generally well understood by most…’ However, some stated that ‘Change of terminology is fine, so long as there is good reason & it doesn't confuse the profession’, which highlights the difficulties that arise when proposing change. When referring to ‘external invasive cervical resorption’ another participant stated ‘Established terminology (as you can tell from my answers, I'm not big on terminology changes unless there is a very good reason for it, especially in a poorly understood area of dentistry such as resorption. Changes in terminology may confuse practitioners even more)’. This may be a common belief carried by practitioners particularly those who are comfortable with the terminology they were taught or use in day‐to‐day clinical practice. Resistance to change is a natural and normal cognitive process used when evaluating new circumstances [24]. Individual attitudes have been reported to be determining factors in change implementation regarding organisational change [24]. As an example, adoption of the latest classification of periodontal conditions in 2017 was met with several concerns, as demonstrated in a study of periodontists in Egypt in 2020 [25], In that study, all participants were aware of the new classification but only 24.2% had adopted it, and only 29.7% considered that the new classification was very good/excellent [25]. It is not unreasonable to expect that there would be some resistance to any new classification but, hopefully, a well‐constructed, clear and simple classification that is consistent with published research findings should help the profession to be more accepting of classifications that have been revised as a result of new knowledge gained through research and clinical practice.

There are several possible limitations to this study. First, there is the possibility that some participants may research their answers while completing the survey. Since some level of application is needed even once appropriate information is sought, this is likely to be a negligible concern, especially considering that the average score for this survey was not very high (52.9%). Second, there may have been a degree of design bias. Perhaps some more information needed to be provided for each question, as in a clinical scenario where dentists are aided by the history and clinical findings, and they may have access to older (or more) radiographs and possibly CBCT scans. However, each type of resorption has specific radiographic features which distinguishes it from the other types [1] and it is not unreasonable to expect dentists to know and recognise these features when assessing radiographs. The clinical information, the extra radiographs and/or CBCT images are useful aids to determine the extent of the resorption, the surfaces involved, and whether the pulp is involved once it has been detected on the initial radiograph. The extra information and other images are also useful aids for determining the management options and the prognosis of the tooth. Third, given this survey was in multiple choice format, it is possible that some dentists ‘guessed’ their answers to most of the questions. Perhaps if there was an answer which read ‘unsure/I don't know’, it may have adequately reflected the confusion rather than participants being forced to select from the options provided.

## 5 Conclusions

Dentists correctly identified approximately half of the types of tooth resorption present on radiographs. Dentists with ‘very good’ self‐reported knowledge had mean scores which were significantly higher than all other groups. The higher mean scores were significantly correlated with male gender, more years since graduation, and specialists. Dentists working in ‘very remote’ areas of Australia had statistically significantly lower mean scores.

Participants preferred the terms ‘external inflammatory resorption’ and ‘external replacement resorption’, but preferences for ‘external invasive resorption’ and ‘external invasive cervical resorption’ were similar. The lack of consensus indicates a need for a standardised universal classification of the different types of tooth resorption.

## Author Contributions

All authors acknowledge significant contribution and agree with the manuscript.

## Ethics Statement

The University of Western Australia's Human Research Ethics Committee approved this research project—approval no. 2021/ET000464.

## Conflicts of Interest

The authors declare no conflicts of interest.

## Supporting information


**File S1:** adj70003‐sup‐0001‐FileS1.pdf.

**File S2:** adj70003‐sup‐0002‐FileS2.pdf.

## Data Availability

The data that support the findings of this study are available from the corresponding author upon reasonable request.
