# Supplementary material for: Dentists' Ability to Identify Tooth Resorption on Radiographic Images and Their Preferences for Terminology
Source: Aust Dent J. 2025 Aug 20;70(4):285–94. doi: 10.1111/adj.70003 (PMC12661130; doi:10.1111/adj.70003)
Supplement: Supplementary file 1 — File S1: adj70003‐sup‐0001‐FileS1.pdf. [file ADJ-70-285-s002.pdf]

Supplementary File 1: Survey Questions

Part 1 – Demographic Questions

Q1.

Please select your gender

- ◯ Male
- ◯ Female
- ◯ Prefer not to disclose
- ◯ Prefer to self describe

Q2.

Q2

What is your age?

Q3.

Where did you complete your primary dental training?

- ◯ Australia
- ◯ New Zealand
- ◯ United Kingdom
- ◯ North America
- ◯ South America
- ◯ Europe
- ◯ Africa
- ◯ Asia
- ◯ Oceania (Excl. Australia and New Zealand)

Q4.

In which year did you complete your primary dental qualification

- Prior to 1951
- 1951-1960
- 1961-1970
- 1971-1980
- 1981-1990
- 1991-2000
- 2001-2010
- 2011-2020

Q5.

Are you a registered dental specialist?

- Yes
- No

Q5a.

What type of registered specialist are you?

- Dento-Maxillofacial Radiology
- Endodontics
- Forensic Odontology
- Oral and Maxillofacial Surgery
- Oral Medicine
- Oral Pathology
- Oral Surgery
- Orthodontics
- Paediatric Dentistry
- Periodontics
- Prosthodontics
- Public Health Dentistry (Community Dentistry)
- Special Needs Dentistry

Q5b.

Where did you complete your specialist training?

- Austraila
- New Zealand
- United Kingdom
- North America
- South America
- Europe
- Africa
- Asia
- Oceania (Excl. Australia and New Zealand)

Q5c.

In which year did you complete your specialist training?

- Prior to 1951
- 1951-1960
- 1961-1970
- 1971-1980
- 1981-1990
- 1991-2000
- 2001-2010
- 2011-2020

Q6.

What is your main area of practice?

- Private
- Public
- Academic
- Retired

Q7.

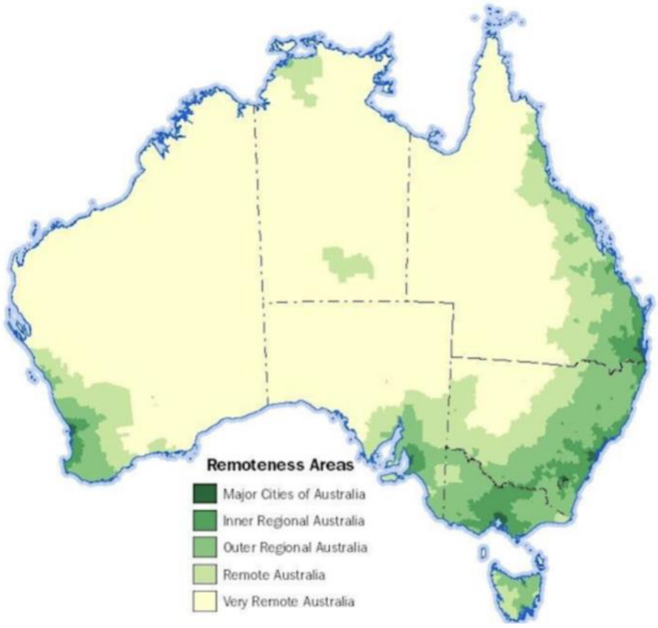

Where do you work?

- RA1: Major Cities of Australia
- RA2: Inner Regional Australia
- RA3: Outer Regional Australia
- RA4: Remote Australia
- RA5: Very Remote Australia

Q8.

How would you rate your knowledge of tooth resorption?

- Low
- Acceptable
- Good
- Very good

Part 2. Radiographs and Questions

Case 1

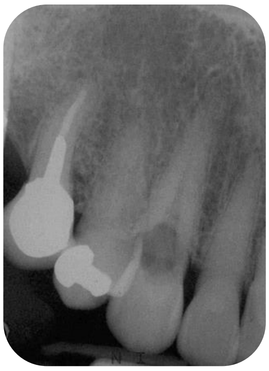

What type of resorption is present?

a) There is no resorption, but caries is present
b) Internal surface resorption
c) Internal inflammatory resorption
d) Internal replacement resorption
e) External invasive resorption
f) External replacement resorption

Case 2

What type of resorption is present?

a) No resorption, but cervical burnout is present
b) No resorption, but caries is present
c) Orthodontic resorption
d) Pressure resorption
e) External inflammatory resorption
f) External invasive resorption

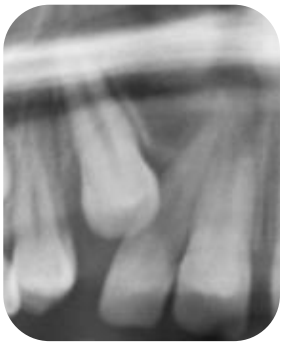

Cropped OPG
September2018

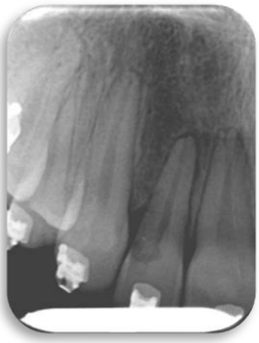

PA August 2020

Case 3

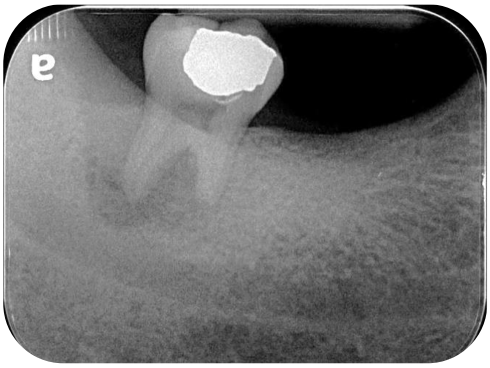

What type of resorption is present?

a) No resorption is present
b) Internal inflammatory resorption
c) External infection-related resorption
d) External replacement resorption
e) Physiological resorption
f) Orthodontic resorption

Case 4

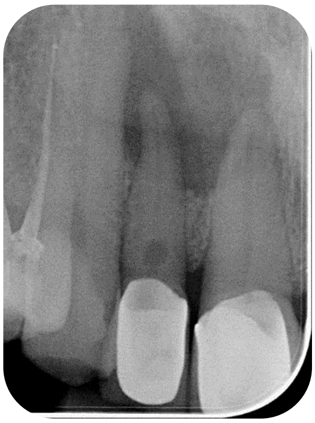

What type of resorption is present?

a) No resorption, but caries is present
b) Internal inflammatory resorption
c) Internal replacement resorption
d) External inflammatory root resorption
e) External invasive root resorption

Case 5

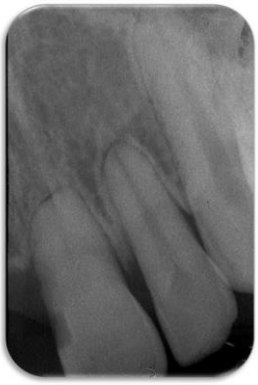

What type of resorption is present?

a) No resorption present
b) Physiological resorption
c) Orthodontic resorption
d) External infection-related resorption
e) External ankylosis-related resorption
f) Idiopathic resorption

Case 6

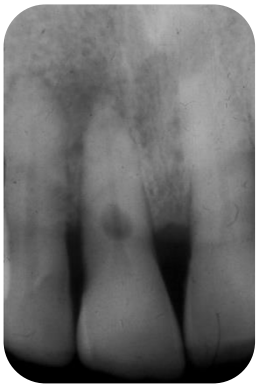

What type of resorption is present?

a) No resorption, but caries is present
b) Internal surface resorption
c) Internal inflammatory resorption
d) External inflammatory resorption
e) External invasive resorption
f) Idiopathic resorption

Case 7

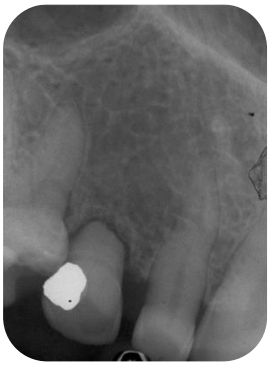

What type of resorption is present?

a) No resorption, but cervical burnout is present
b) Physiological resorption
c) Pressure resorption
d) Orthodontic resorption
e) External invasive resorption
f) Idiopathic resorption

Case 8

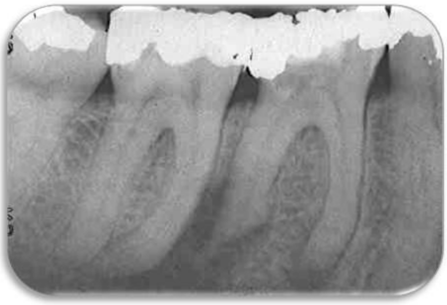

What type of resorption is present?

a) No resorption, but cervical burnout is present
b) Pressure resorption
c) Orthodontic resorption
d) External inflammatory resorption
e) External invasive resorption

Case 9

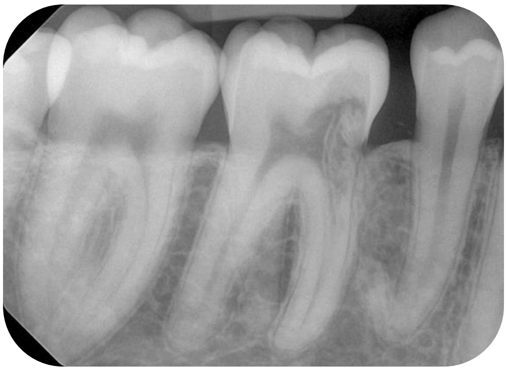

What type of resorption is present?

a) No resorption, but caries is present
b) Pressure resorption
c) Orthodontic resorption
d) External inflammatory resorption
e) External invasive resorption
f) External replacement resorption

Case 10

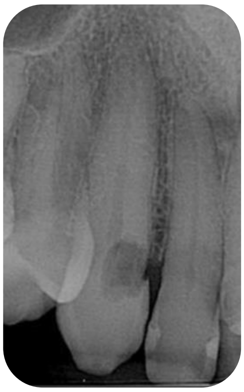

What type of resorption is present?

a) No resorption, but cervical burnout is present
b) No resorption but caries is present
c) Internal inflammatory resorption
d) External infection-related resorption
e) External invasive resorption
f) Idiopathic resorption

Case 11

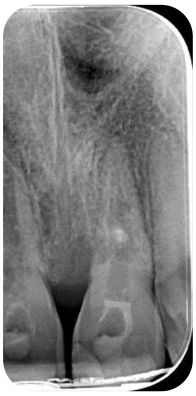

What type of resorption is present?

a) There is no resorption
b) Internal surface resorption
c) Internal inflammatory resorption
d) Internal replacement resorption
e) External invasive resorption
f) External ankylosis-related resorption

Case 12

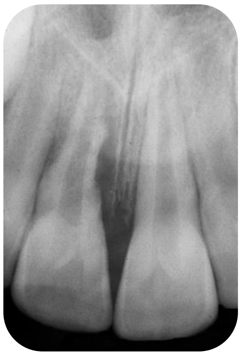

What type of resorption is present?

a) No resorption is present
b) Internal inflammatory resorption
c) External infection-related resorption
d) External replacement resorption
e) Physiological resorption
f) Orthodontic resorption

Part 3. Preferred Terms

There is no consistency within the literature concerning the terminology used regarding the various forms of tooth resorption. In each of the following questions, the terms listed are commonly used in the literature for the same clinical condition. The questions ask you to nominate your preferred name for several types of resorption.

1

Which term do you prefer and why?

○ External inflammatory resorption

○ External infection-related resorption

2

Which term do you prefer and why?

○ Replacement resorption

○ Ankylosis-related resorption

3

Which term do you prefer and why?

- ◯ External invasive resorption

- ◯ External cervical resorption

- ◯ External invasive cervical resorption

Correct Answers for Part 2:

Question Number	Correct Answer
1	**E**
2	**D**
3	**C**
4	**B**
5	**C**
6	**E**
7	**B**
8	**D**
9	**E**
10	**E**
11	**F**
12	**C**
