# Supplementary material for: Dentists' Ability to Identify Tooth Resorption on Radiographic Images and Their Preferences for Terminology
Source: Aust Dent J. 2025 Aug 20;70(4):285–94. doi: 10.1111/adj.70003 (PMC12661130; doi:10.1111/adj.70003)
Supplement: Supplementary file 2 — File S2: adj70003‐sup‐0002‐FileS2.pdf. [file ADJ-70-285-s001.pdf]

Supplementary Table 1

Summaries (and numbers) of the reasons provided by participants for their choices of the different terms for the three types of resorption, plus examples of specific reasons for each type of resorption

EXTERNAL INFLAMMATORY RESORPTION					
**No Reason (67)**	**Familiar (71)**	**Descriptive (98)**	**Self-Explanatory (28)**	**Generalised (63)**	**Other (28)**
Left blank/both/ preferred (56)	Used or taught at university (49)	Accurate/specific/descriptive to the disease process (mechanism/physiology/ pathology) (87)	Easier to understand/apply/ self-explanatory/clearer/makes sense (13)	More inclusive/broader/all-encompassing/general (37)	Reason unclear/ incomplete/technically incorrect (9)
Unsure (4)	Seen more/more common/heard more/most familiar (17)	Describes the process not the cause/source (3)	Concise/simpler/shorter (15)	May not be infection-related (18)	Difficult to diagnose cause (difficult to diagnose from radiograph) (8)
No preference (5)	Have not heard of the other (5)	It is inflammation - Infection may or may not be present/related (6)		May be related to trauma (3)	Sounds better/sounds better to patients (3)
Neither (2)		Inflammation encompasses infection/Infection = inflammation (2)		Does not exclude non-infection causes (5)	Consistent with literature/standardising literature/medically sound (6)
					Other (2)

Some examples of reasons provided for EXTERNAL INFLAMMATORY RESORPTION

No Reason	Familiar	Descriptive	Self-Explanatory	Generalised	Other
**“**This term”<br><br>“To be honest I don't know the literature well enough to prefer a term”<br><br>“Preferred”<br><br>“I prefer neither as I know nothing about them.”<br><br>“I don't like either. This survey makes me think I have been using the wrong terms”	“Preferred because this is what I was taught at dental school”<br><br>“I prefer this one as I have not heard of the other”<br><br>“It’s the term I’m most familiar with”<br><br>“Seen more commonly”	“It is osteoclast activity (RANKL/OPG) that causes resorption not a specific bacteria”<br><br>“I prefer to use this term as inflammation causes resorption as a chronic process not infection which relates to acute signs and symptoms.”<br><br>“inflammation seems a more accurate description of the pathologic process”<br><br>“More in line with Endodontic lesions. We don’t say “infection related peri-apical periodontitis”, it is purely acute or chronic apical periodontitis. By this same premise, inflammation is assumed with external inflammatory rather than “infection related””<br><br>“Describes the physiological response as opposed to a lay sounding description”	“Self-explanatory”<br><br>“I prefer this term due to its simplicity”<br><br>“Brevity”<br><br>“More succinct”<br><br>“Shorter name”<br><br>“It’s shorter to write”	“This term feels more inclusive and language-appropriate.”<br><br>“Less specific term as the alternative option indicates the inflammatory reaction is infection-related.”<br><br>“Broad non-specific term”<br><br>“No all inflammation is infection”<br><br>“Inflammation encompasses infection”	“The resorption is not due to an infection”<br><br>“Radiographic report is not a diagnosis but an observation”<br><br>“I have now realised how little I know about the terms and types of resorption”<br><br>“Is this process inflammatory or infection related? Prefer the name that reflects the pathophysiology”<br><br>“Because it is consistent with the remaining categories of 11 resorptions. cf. "infection related" is a category used in the new but incomplete clarification system that has been proposed.”<br><br>“Most of the time there is resorption and no infection”

EXTERNAL INFECTION-RELATED RESORPTION					
**No Reason (10)**	**Familiar (3)**	**Descriptive (21)**	**Self-Explanatory (25)**	**Aetiology Specified (36)**	**Other (4)**
Left blank/this/name repeated (10)	Taught at dental school (1)	More/better descriptive/precise/ specific (18)	Easier to understand/apply/ self-explanatory/clearer/makes sense (16)	Cause/reason for resorption identified (31)	Intuitive (1)
	More common (2)	Inflammatory too vague (3)	Makes sense (2)	Cause and effect/treatment outlined (5)	Sounds better (1)
			Easier to explain to patients (7)		Other (2)

Some examples of reasons provided for EXTERNAL INFECTION-RELATED RESORPTION					
**No Reason**	**Familiar**	**Descriptive**	**Self-Explanatory**	**Aetiology Specified**	**Other**
"I like this one because"<br><br>"This"<br><br>"External resorption"	"This term might have been what I was taught to use during university"	"It is more directed. Inflammatory could mean several different things - more vague."<br><br>"Preference because it explains how the resorption occurred"<br><br>"Reiterates that there is an infective process involved in the pathology"	"External resorption due to infection is easier to explain to patients"<br><br>"Much easier to relate to and simple sounding"<br><br>"Prefer infection related - conveys the severity of the damage to patient in language they most likely understand. They know infection is not good and needs treatment. inflammation in their minds, can often be treated less invasively"	"This specifies the reason for resorption and automatically the appropriate treatment"<br><br>"This explains the aetiology better"<br><br>"This describes both the etiological factor and the location/root surface invoiced by the resorptive defect. The term also indicates the treatment required for managing the cause - ie. appropriate management of the intracanal infection should predictably stop the progression of the resorptive process."	"I don't know much about resorption but this seems more intuitive"<br><br>"because it tells the pt what is causing it"<br><br>"Sounds more correct, although the other is more historically familiar"

EXTERNAL REPLACEMENT RESORPTION					
**No Reason (67)**	**Familiar (63)**	**Descriptive (93)**	**Self-Explanatory (30)**	**Generalised (12)**	**Other (35)**
Blank/Replacement resorption/This one/Preferred/Yes (57)	Taught at dental school (45)	Ankylosis can occur without replacement resorption (6)	Simpler/clearer/easier to understand (12)	More generalized (9)	Not all replacement resorption will have ankylosis/replacement resorption results in ankylosis (17)
Both/either/ interchangeable (3)	Familiar (7)	Better descriptor/process/ physiology/comprehensive/ specific (77)	Easier for patients to understand (7)	Ankylosis too specific (3)	Consistent with literature (3)
Neither (1)	More common/seen more (9)	Describes what you see on the x-ray/visualise (radiograph) (8)	Makes more sense (9)		Ankylosis can only be confirmed clinically/ histologically (5)
Unsure/No idea (6)	Never heard of ankylosis-related resorption before (2)	Resorption and ankylosis are two separate conditions although related (2)	Shorter (2)		Other (10)

Some examples of reasons provided for EXTERNAL REPLACEMENT RESORPTION

No Reason	Familiar	Descriptive	Self-Explanatory	Generalised	Other
“No idea what either of these terms are.”<br><br>“Not too sure to be honest”<br><br>“Both are appropriate, just explaining the underlying cause.”<br><br>“Prefer neither as not familiar with either”	“Never heard of ankylosis related resorption before”<br><br>“Prefer this one as have just seen it used more often in literature I’ve come across.”<br><br>“This because I was taught this at dental school”	“I have no idea what ankylosis related resorption is. I suppose it means the same thing? but from a histological point of view, replacement sounds right.”<br><br>“This one, as Ankylosis doesn’t always have replacement with the bone.”<br><br>“The term is more consistent with the resorption process”<br><br>“I prefer this term as it doesn’t make it sound like ankylosis “causes” replacement resorption”<br><br>“Describes what you see on the x-ray”	“Replacement resorption is a name that you can even tell the patient and they can understand the name, however ankylosis is not as usual.”<br><br>“Easier to understand”<br><br>“Easier to understand and relate to patient.”<br><br>“Shorter term”<br><br>“Less convoluted”	“Is a more generalized term which is not limited to immobility (ankylosis)”<br><br>“This term. Ankylosis is too specific”	“There can be hard tissue replacing tooth structure without ankylosis”<br><br>“Consistent with established classification system.”<br><br>“Ankylosis requires a clinical exam to be certain”<br><br>“Not all replacement will have ankylosis”

EXTERNAL ANKYLOSIS-RELATED RESORPTION					
**No Reason (17)**	**Familiar (7)**	**Descriptive (30)**	**Self-Explanatory (34)**	**Aetiology Specified (17)**	**Other (13)**
Blank/ Preferred/ No reason/ no opinion/ name repeated (13)	Taught at dental school (2)	Descriptive of process/direct/specific (19)	Simpler/clearer/easier to understand/explain (22)	Cause/reason specified/aetiology clear (17)	Sounds better (2)
Unsure/don't know (3)	Familiar (2)	Clear that ankylosis must be present/outcome/ effect (8)	Makes sense/intuitive (8)		Related to well-known term of ankylosis (2)
Any/either (1)	Read/seen more (2)	Describes what you would see (3)	Less confusing (2)		Trauma history (2)
	Haven't heard of the other (1)		More information is given if one did not understand replacement resorption/associated with ankylosis (2)		Other (7)

Some examples of reasons provided for EXTERNAL ANKYLOSIS-RELATED RESORPTION					
**No Reason**	**Familiar**	**Descriptive**	**Self-Explanatory**	**Aetiology Specified**	**Other**
"I prefer this one"<br><br>"Unsure"<br><br>"I don't know"<br><br>"Any"	"I was taught to use this term during university"<br><br>"More familiarity with the term"	"Possibly better because it acknowledges that ankylosis must be present as the precursor to and the cause of any replacement resorption"<br><br>"This term describes what you would see"<br><br>"Visual prompt"<br><br>"More descriptive"	"Term is easier to understand."<br><br>"Easier to explain to patients than describing the microscopic detail of osteoclasts, inflammatory mediators etc"<br>"Although I never have used the term "ankylosis-related resorption" I feel that the term just makes more sense than replacement resorption."<br><br>"This would help most GP clinicians associate the ankylosis with the pathology."	"It's specific and directly relates to the cause of the pathological lesion"<br><br>"Prefer this one, as this one also possible highlights the cause of the resorption i.e. caused due to ankylosis"<br><br>"Prefer this because it describes the aetiology/source of resorption"	"Related to well-known term of ankylosis"<br><br>"Ankylosis is a term I use often with extractions"<br><br>"More scientific<br><br>I prefer this only because I know something about ankylosis. At this point I feel like I should have put my replacement knowledge as "low""<br><br>"Too limiting?"

EXTERNAL INVASIVE RESORPTION					
**No Reason (33)**	**Familiar (17)**	**Descriptive (29)**	**Self-Explanatory (18)**	**Generalised (55)**	**Other (6)**
Blank/This/Name repeated/preferred (29)	Taught at dental school (14)	Specific/descriptive/ detailed/process/ comprehensive / accurate (29)	Simpler/clearer/easier to understand/obvious/direct/ Less confusion/easy to remember (10)	Broader (less specific)/doesn't restrict/ not limited/overarching term (15)	Other (6)
Not sure (2)	Seen more/used to this term (3)		Makes sense (2)	Can occur anywhere, not just cervical (40)	
All are fine/No preference (2)			Shorter/concise (2)		
			Easier for the patient to understand (4)		

Some examples of reasons provided for EXTERNAL INVASIVE RESORPTION

No Reason	Familiar	Descriptive	Self-Explanatory	Generalised	Other
"This one"<br><br>"Preferred"<br><br>"Not sure"	"Only definition I am familiar with"<br><br>"What I was taught in dental school"<br><br>"Already known"	"It is specific, not too long and directly relates to the cause of the pathological lesion"<br><br>"Denotes a clear route and scenario"<br><br>"I Prefer this term because it explains how the soft tissue invades into the tooth causing the resorption"<br><br>"Description in name hard to beat Immediately less confusion"<br>"Of the three terms, this is most descriptive. Resorption may not always occur at the cervix (?)."	"Most simple form, easy to explain and interpret."<br><br>"Easier to understand and relate to patient."<br><br>"This is the name I would prefer, for the same reason as the words are more simple for the patient to understand. "<br><br>"This would be preferred, as it's more simple"	"This can occur in regions other than cervical therefore it may be more useful to have one overarching term. However the two entities have been separated in different literature"<br><br>"Potential to happen elsewhere apart from the cervical region"<br><br>"Not limited to the cervical region of the tooth."<br><br>"Not always at the cervical third if the root and depends on where the epithelial attachment is."<br><br>"Because it doesn't specify the location which means it is a broader term"	"Prefer external invasive cervical resorption"<br><br>"Appears more serious"<br><br>"Didn't know they meant the same thing."<br><br>"I have now realised how little I know about the terms and types of resorption"<br><br>"This Depends on how you define where cervical starts and stops"<br><br>"Cervical related to burnout"

EXTERNAL CERVICAL RESORPTION					
**No Reason (21)**	**Familiar (22)**	**Descriptive (13)**	**Self-Explanatory (16)**	**Location Specified (34)**	**Other (8)**
Blank/preferred/this/ name repeated (19)	Taught at dental school (15)	Specific/detailed/describes process/precise (13)	Simpler/clearer/easier to understand (12)	Location/origin specified (34)	Other (8)
Unsure (2)	Familiar (1)		Easier to explain to patients (1)		
	Seen more/heard more/used more (5)		Makes sense (1)		
	Only one I've heard of (1)		Easier to remember (2)		

Some examples of reasons provided for EXTERNAL CERVICAL RESORPTION					
**No Reason**	**Familiar**	**Descriptive**	**Self-Explanatory**	**Location Specified**	**Other**
"Preferred"<br><br>"This"<br><br>"Unsure"	"This term was used in the literature during my dental training."<br><br>"This is what we were taught"<br><br>"I hear this term more often"	"Preferred, good descriptor"<br><br>"This one as I am most familiar with it and accurately describes the process."<br><br>"This is most specific"	"Easier to understand"<br><br>"More simple and concise"<br><br>"Simple and what I use"	"As this refers to the location of the resorption"<br><br>"Generally located in the cervical region. Don't like invasive sounds cancerous"<br><br>"Location clearer in title."<br><br>"Describes the origin"	"Best middle descriptor"<br><br>"Most resorptions are invasive anyway"<br><br>"More generally applicable "

EXTERNAL INVASIVE CERVICAL RESORPTION					
**No Reason (32)**	**Familiar (32)**	**Descriptive (86)**	**Self-Explanatory (11)**	**Location Specified (32)**	**Other (4)**
Blank/this/preferred/ name repeated/yes (32)	Taught at dental school (18)	Descriptive/detailed/process/ specific/Comprehensive/ complete (86)	Simpler/clearer/easier to understand/obvious/direct Less confusing (9)	Location/origin /site identified (32)	Other (4)
	Familiar (6)		Logical/makes sense (2)		
	Seen more/more common (8)				

Some examples of reasons provided for EXTERNAL INVASIVE CERVICAL RESORPTION					
**No Reason**	**Familiar**	**Descriptive**	**Self-Explanatory**	**Location Specified**	**Other**
"This"<br><br>"Prefer this"<br><br>"Yes"	"Heard this term more commonly"<br><br>"EICR has always been a well-accepted acronym"<br><br>"Established terminology. (As you can tell from my answers I'm not big on terminology changes unless there is a very good reason for it, especially in a poorly understood area of dentistry, such as resorption. Changes in terminology may confuse practitioners even more)."	"EICR is better suited as this term covers the resorption characteristics appropriately."<br><br>"I think this term most accurately describes this particular form of pathosis."<br><br>"This seems to be the all-encompassing term, simplifying how many overall categories resorption defects should be classified into"<br><br>"Gives most complete description. I am comfortable with all these terms."<br><br>"This term shows that characteristic and nature seen of this resorption"<br><br>"Encompasses all key aspects of the condition"<br><br>"This one seems the most specific"	"Describes it more clearly"<br><br>"Most logical"<br><br>"Makes sense"	"More specific to location"<br><br>"Location"<br><br>"More closely describes location"<br><br>"Relates to position and aggressiveness"<br><br>"Specifies then location and type with most detail"	"Best of both world"<br><br>"I thought this was the norm"<br><br>"More inclusive"<br><br>"Nailed it to the tea"
